# Vision-targeted health related quality of life in older adults: patient-reported visibility problems in low luminance activities are more likely to decline than daytime activities

**DOI:** 10.1186/s12886-016-0274-5

**Published:** 2016-07-07

**Authors:** Cynthia Owsley, Gerald McGwin

**Affiliations:** Department of Ophthalmology, School of Medicine, University of Alabama at Birmingham, Birmingham, AL 35294-0009 USA; Department of Epidemiology, School of Public Health, University of Alabama at Birmingham, Birmingham, AL 35294-0022 USA

**Keywords:** Aging, Vision, Quality of life, Questionnaire, Visual activities, Low luminance, Night vision

## Abstract

**Background:**

Commonly used vision-targeted health-related quality of life questionnaires almost exclusively focus items on vision under daytime conditions. Older adults even when in good eye health frequently report experiencing vision problems at night and under low environmental light levels, and psychophysical studies also document these visibility problems. Here we compare the progression of self-reported low luminance visibility problems and self-reported visibility problems under daytime conditions in older adults.

**Methods:**

Trained interviewers administered two questionnaires to older adults in normal eye health: the National Eye Institute Visual Function Questionnaire – 25 (NEI VFQ-25) where items are almost entirely focused on difficulties in daytime activities, and the Low Luminance Questionnaire (LLQ) where items are focused on difficulties seeing at night and under low luminance conditions. The following visual functions were also measured: visual acuity, low luminance visual acuity, low luminance deficit, contrast sensitivity, light sensitivity in the macula, and rod-mediated dark adaptation. The protocol was repeated 3 years later.

**Results:**

Scores on the NEI VFQ-25 composite and its subscales were unchanged between baseline and 3-year follow-up, whereas scores on the LLQ composite and 5 of 6 subscales significantly decreased (corresponding to less functionality) at the 3-year follow-up. Participants were more likely to display a ≥ 5 point decrease on the LLQ composite than on the NEI VFQ-25 over 3 years. Visual functional tests were largely unrelated to changes in NEI VFQ-25 and LLQ scores from baseline to follow-up.

**Conclusions:**

Older adults’ vision-targeted quality of life as measured by questionnaire is more likely to exhibit a practically significant decrease over 3 years using a questionnaire that focused on low luminance activities (LLQ) than one focused on daytime activities (NEI VFQ-25). That the results of visual functional testing did not correspond to older adults’ decline in self-reported problems in low luminance activities emphasizes the importance of questionnaires in understanding visual difficulties from the patients’ own perspective.

## Background

Older adults experience aging-related challenges in the visual activities of daily living, even in the absence of the common eye conditions and diseases of later adulthood (e.g., cataract, age-related macular degeneration (AMD), glaucoma, diabetic retinopathy). They frequently report experiencing vision problems at night and under low environmental light levels. For example, they cite difficulty with night driving and often avoid it [1–3] and report task difficulties under low illumination (e.g., reading a menu in a dimly lit restaurant) [4]. Psychophysical studies confirm that older adults, even when free of significant ocular conditions, tend to exhibit decreased scotopic and mesopic light sensitivity, contrast sensitivity, and acuity, as compared to younger adults [5–10].

It is thus concerning that commonly used vision-targeted health-related quality of life questionnaire instruments designed for use in the older adult population almost exclusively focus items on vision under photopic (day-time) conditions, such as the National Eye Institute Visual Function Questionnaire (NEI VFQ-25,) [11] among others [12, 13]. Thus, these questionnaire instruments do not adequately address one of the major vision problem areas cited by older adults, activities at lower ambient light levels. In response to a need for a questionnaire focused on low luminance content, a questionnaire was recently developed specifically targeted at this content; the Low Luminance Questionnaire (LLQ) is a 32-item questionnaire designed for use with older adults and has established content and construct validity and test-retest reliability [14, 15] The LLQ is accessible at http://www.uab.edu/medicine/ophthalmology/images/Research/Low%20Luminance.pdf). It has six subscales: driving, extreme lighting, mobility, emotional distress, general dim lighting, peripheral vision.

Even though considerable research has shown that older adults’ vision problems are exacerbated under low luminance as measured psychophysically and by self-report, it remains to be determined whether self-reported low luminance visibility problems progress more rapidly over time in older adults as they age as compared to visibility issues they cite under daytime conditions. Here we examine whether responses on the LLQ display larger decreases over 3 years in older adults free of significant ocular conditions, as compared to responses on the NEI VFQ-25, which is largely focused on daytime activities. We also examined whether decreases in questionnaire scores were related to visual function at baseline and change in visual function over a 3-year period of follow-up.

## Method

The protocol (#F080205001) was approved by the Institutional Review Board of the University of Alabama at Birmingham (UAB) and followed the tenants of the Declaration of Helsinki. Informed consent was obtained from all participants after the nature and purpose of the study was described. This study made use of the sample assembled for the Alabama Study on Early Age-Related Macular Degeneration (ALSTAR), a prospective study of older adults in normal macular health at baseline [16, 17]. As described previously, participants were recruited from two primary care ophthalmology practices in the Callahan Eye Hospital at UAB. Eligibility criteria were as follows: (1) age ≥ 60 years old; (2) normal macular health in both eyes as determined by 3-field digital stereo-fundus photos (Carl Zeiss Meditec 450 Plus camera, Dublin, CA) evaluated by an experienced grader masked to other study variables. Each eye’s grade had to be 1 in the Age-Related Eye Disease Study (AREDS) 9-step classification system [18], indicating normal macular health. (3) No previous diagnoses of glaucoma, other retinal conditions, optic nerve conditions, corneal disease, diabetes, Alzheimer’s disease, Parkinson’s disease, brain injury, other neurological or psychiatric conditions as revealed by the medical record or by self-report.

A baseline visit consisted of the following. Participants provided information on demographic characteristics (age, gender, race/ethnicity). The NEI VFQ-25 and the LLQ were interviewer administered. Both of these instruments were developed using classical test theory, [19] and we elected to score each instrument using recommended scoring instructions by their developers [11, 14] The NEI VFQ-25 and the LLQ composite and subscale scores are computed using the same method, that is, by scaling individual item responses from 0 to 100, where 100 represents the highest functional level and 0 the lowest, and then averaging the individual items. Other options for scoring these types of questionnaires include a collection of measurement models referred to as item response theory [19]. General cognitive status was estimated by the Mini-Mental State Examination (MMSE) [20]; scores less than 24 indicated cognitive impairment.

Several visual function tests were also administered; tests were administered for each eye unless otherwise noted. The eye with better visual function was used in analyses. Best-corrected visual acuity for each eye was assessed via the Electronic Visual Acuity tester [21] (EVA; JAEB Center, Tampa FL) under photopic conditions (100 cd/m^2^) and expressed as the logarithm of the minimum angle resolvable (logMAR). The EVA was also used to assess low luminance visual acuity for each eye, with participants viewing letters through a 1.5 log unit neutral density filter [22]. The filter reduced background luminance to 3.1 cd/m^2^. To determine how much logMAR decreased under conditions of the lower light level as compared to the photopic (100 cd/m^2^) assessment, we defined a decrease in visual acuity under low luminance by the increase in logMAR (referred to as the “low luminance deficit” [22]). Contrast sensitivity for each eye was estimated by the Pelli-Robson chart [23] (Precision Vision, La Salle, IL) with mean luminance of 100 cd/m^2^, the letter-by-letter scoring method [24], and expressed as logarithm of sensitivity. Macular light sensitivity for each eye was assessed using the Humphrey Field Analyzer (Carl Zeiss Meditec, Dublin, CA) and the 24-2 SITA standard protocol, as previously described [25]. Sensitivities for 16 test targets within a 9° × 9° macular region were averaged, and expressed as decibels (dB). Rod-mediated dark adaptation was measured using a computerized dark adaptometer as described previously [16, 17]. Test targets with a diameter of 2° were positioned at 5° on the inferior vertical meridian (superior to the fovea on the retina). Following a photobleach exposure (equivalent ~83 % bleach), sensitivity was measured at 30-s intervals for 20 min following bleach offset. Dark adaptation time was defined by the rod intercept, the time in minutes needed by the participant to reach a criterion sensitivity value in the latter half of the second component of rod recovery. Due to time constraints in the protocol visit, dark adaptation was measured in one eye only.

Administration of the NEI VFQ-25, LLQ, and visual function testing was repeated 3 years after the baseline visit. The only exception was measurement of macular light sensitivity, which was not repeated at the 3-year follow-up due to time constraints in the study protocol. The eye with better visual function was used in analyses.

Baseline and 3-year follow-up NEI-VFQ and LLQ composite and subscale scores were compared using paired t-tests. Unpaired t-tests were used to compare participants whose LLQ composite did and did not decrease by five or more points with respect to visual function variables. A similar analysis was conducted for the NEI-VFQ composite. P-values less than 0.05 (two-tailed) were considered statistically significant.

## Results

There were 365 participants in the ALSTAR study in normal eye health who completed both the baseline and follow-up LLQ and NEI VFQ-25. The vast majority of the sample were in their 60s or 70s (97.5 %) and were white of non-Hispanic origin (94.5 %) (Table 1). Approximately 2/3 of the sample was women. In terms of cognitive status, 98.1 % (358 of 365) had MMSE scores in the non-cognitively impaired range (24–30).

**Table 1:** Demographic and cognitive status characteristics of the sample, *N* = 365

Characteristic	*n* (%)
Age, years, *n* (%)	
60–69	246 (67.4)
70–79	110 (30.1)
80–89	9 (2.5)
Gender, *n* (%)	
Men	123 (33.7)
Women	242 (66.3)
Race/ethnicity	
White, non-Hispanic	345 (94.5)
African American	16 (4.4)
Other	4 (1.1)
Cognitive status, MMSE score	
28–30	281 (77.0)
26–27	57 (15.6)
24–25	20 (5.5)
≤ 23	7 (1.9)

Table 2 shows the mean and standard deviation for the NEI VFQ-25 composite and subscale scores at both baseline and the 3-year follow-up, along with the corresponding change in score (baseline minus follow-up score). The NEI VFQ-25 composite and all subscales were unchanged between baseline and 3-year follow-up (except for ocular pain which increased slightly). Table 3 provides the analogous information for the LLQ. The LLQ composite and 5 of 6 LLQ subscales decreased between baseline and 3 years later (all *p* < 0.007). The exception was the general dim lighting subscale (*p* = 0.0579), which did not reach statistical significance.

**Table 2:** NEI-VFQ Composite and Subscales scores at baseline and follow-up for participants in normal macular health group and the change in scores over this 3-year period (*N* = 365)

	Normal macular health at baseline ^a^			
NEI-VFQ subscales	Baseline score	3-year follow-up score	Difference ^a^	*P*-value
	M (SD)			
Composite	93.9 (5.5)	93.9 (5.9)	0.0	0.9875
General health	73.9 (21.7)	72.8 (21.7)	1.1	0.2724
General vision	83.7 (11.2)	82.5 (11.7)	1.2	0.0781
Near vision	92.3 (11.1)	92.7 (11.4)	−0.5	0.4984
Distance vision	93.0 (8.9)	92.8 (10.0)	0.2	0.6675
Driving	89.7 (10.9)	90.1 (10.6)	−0.3	0.5635
Peripheral vision	94.9 (11.9)	94.7 (12.5)	0.3	0.6773
Color vision	97.9 (8.85	98.4 (7.4)	−0.3	0.3699
Ocular Pain	90.3 (12.0)	91.8 (11.7)	−1.4	0.0221
Vision-specific				
Role difficulties	96.0 (9.8)	95.7 (10.6)	0.3	0.6139
Dependency	99.4 (3.5)	99.0 (5.0)	0.4	0.1419
Social functioning	98.7 (5.0)	99.1 (4.5)	−0.3	0.3565
Mental health	94.5 (8.1)	94.0 (10.5)	0.5	0.3654

^a^ Difference is baseline score minus follow-up score

**Table 3:** LLQ Composite and Subscales scores at baseline and follow-up for participants in normal macular health group and the change in scores over this 3-year period (*N* = 365)

	Normal macular health at baseline ^a^			
LLQ subscales	Baseline score	3-year follow-up score	Difference ^a^	*P*-value
	M (SD)			
Composite	91.4 (8.8)	89.7 (10.3)	1.7	<0.0001
Driving	85.0 (19.0)	81.8 (22.8)	3.1	0.0009
Extreme lighting conditions	87.2 (11.2)	85.0 (13.5)	2.2	0.0005
Mobility	96.0 (7.5)	94.6 (8.9)	1.4	0.0014
Emotional distress	97.6 (6.2)	96.3 (7.9)	1.2	0.0023
General dim-lighting	91.5 (11.4)	90.5 (11.8)	1.0	0.0579
Peripheral vision	93.4 (12.3)	91.4 (14.8)	2.0	0.0065

^a^ Difference is baseline score minus follow-up score

Although the scores for the LLQ composite and 5 subscales decreased over 3 years, the decrease on average was small, approximately 1 to 3 points, leading one to question its clinical or practical significance. Thus we defined a clinically significant decrease in LLQ score to be a decrease of ≥ 5 points from baseline to 3-year follow up. Our rationale was based on an accepted approach to determining minimally important changes in health-related quality of life, namely defining it as approximately half a standard deviation [26]. The standard deviation for the LLQ composite was approximately 10, and thus we defined our minimally important difference as 5 points on the LLQ. Eighty-five of 365 (23.3 %) participants had ≥ 5 point decrease in the LLQ composite between baseline and follow-up (Table 4), which is significantly larger than the 41 participants (11.2 %) who had ≥ 5 point decrease on the NEI VFQ-25 composite over the same time period, *p* < 0.0001. The seven individuals with MMSE scores < 24 (indicating cognitive impairment) had greater decreases on both the NEI VFQ-25 and LLQ at the 3-year follow-up, as compared to those with MMSE scores ≥ 24, although this association did not reach statistical significance for the LLQ (*p* = 0.0024 and *p* = 0.0801, respectively). The pattern of results in the tables did not change when these seven persons were dropped from the analysis.

**Table 4:** Percentage of participants having a ≥ 5 point decrease in the NEI VFQ-25 composite as compared to the LLQ composite over 3 years

	Composite decreased by ≥ 5 points	Composite did not decrease by ≥ 5 points	*p*-value
	*n* (%)		
NEI VFQ-25	41 (11.2)	324 (88.8)	<0.0001
LLQ	85 (23.3)	280 (76.7)	

We examined whether visual function (visual acuity, contrast sensitivity, macular light sensitivity, low luminance deficit, rod-mediated dark adaptation) at baseline and change in these visual functions from baseline to follow-up were associated with participants who had ≥ 5 point decrease in LLQ composite score at 3 years (Table 5). No aspect of visual function tested at baseline or its change score was related to those who had ≥ 5 point LLQ composite drops. This was also the case for the NEI VFQ-25 with one exception; a 0.10 logMAR decline in visual acuity was associated with a ≥ 5-point decrease on the NEI VFQ-25, as compared to those who declined < 5 points.

**Table 5:** Vision variables stratified by participants with a ≥ 5 point decrease in the LLQ composite from baseline to 3-year follow-up versus those whose scores did not decrease by ≥ 5 points

	LLQ composite decreased by ≥ 5 points *N* = 85	LLQ composite did not decrease by ≥ 5 points *N* = 280	*p*-value	NEI VFQ-25 composite decreased by ≥ 5 points *N* =	NEI VFQ-25 composite did not decrease by ≥ 5 points *N* =	*p*-value
	Mean (standard deviation) unless otherwise noted					
Visual acuity at baseline (logMAR)	−0.02	−0.02	0.9908	0.00	−0.02	0.1196
Change in visual acuty ^a^	−0.06	−0.04	0.4341	−0.10	−0.04	0.0145
Contrast sensitivity, log sensitivity	1.63	1.65	0.1482	1.65	1.64	0.7051
Change in contrast sensitivity	0.05	0.04	0.7315	0.06	0.04	0.3192
Light sensitivity, dB ^b^	30.4	30.6	0.3264	30.5	30.6	0.8041
Low luminance deficit	0.30	0.29	0.5884	0.31	0.29	0.2780
Change in low luminance deficit	−0.05	−0.04	0.7105	−0.08	−0.04	0.1039
Rod-mediated dark adaptation, rod intercept (minutes)	10.45	10.10	0.4050	10.1	10.2	0.8394
Change in rod-mediated dark adaptation	−1.26	−0.85	0.3121	−1.8	−0.8	0.0832

^a^ Change in visual function variables defined as the value at baseline minus the value at the 3-year follow-up

^b^ Light sensitivity was only measured at baseline

## Discussion

Our results suggest that a vision-targeted health-related quality of life questionnaire whose content is focused on night-time and low luminance visual activities is more likely to show a decline over time in older adults than a questionnaire that focuses on daytime activities. Older adults’ composite scores were more likely to have a practically significant decrease over 3 years using a questionnaire that focused on low luminance activities (LLQ) than one focused on daytime activities (NEI VFQ-25). In addition, scores on five of six subscales of the LLQ significantly declined 3 years later, however none of the twelve subscales of NEI VFQ-25 declined over the same period. Our results imply that the use of questionnaires in clinical vision research on older adults that exclusively target daytime visual activities are likely to miss certain types and the extent of visibility problems older adults experience in daily life. It is noteworthy that this pattern of results emerged even in older adults in normal eye health, free of the common eye conditions of aging. Common ageing-related eye conditions such as AMD and glaucoma are associated with visual deficits under mesopic and scotopic conditions, which are more severe than what would be expected through aging alone [14, 27–29]. It remains to be determined whether changes in the LLQ are associated with patients’ perceptions about the worsening of low luminance vision in these conditions, as they progress.

This greater tendency for older adults’ self-report activity problems under low luminance than in photopic conditions is consistent with psychophysical reports that older adults’ visibility problems are accentuated under mesopic and scotopic conditions, as compared to younger adults [7–9, 30]. However, in this study there was no association between psychophysically measured deficits and self-reported deficits as reported on the LLQ. Those persons who had worse visual function at baseline or had more visual decrease over 3 years, were not more likely to be those who had larger decreases on the LLQ over the 3 year period. This study focused on older adults in normal health, and it is possible that psychophysical testing is not sensitive enough to quantitate the low luminance difficulties in everyday life older adults in good eye health experience subjectively.

## Conclusions

How patients view their own visual task difficulties and the emotional consequences these challenges present is an appropriate part of understanding the impact of eye conditions and aging on everyday life. Patient reported outcomes, such as questionnaires on vision-targeted health-related quality of life, are an essential part of evaluating the impact of ophthalmic interventions to slow the progression of age-related eye conditions such as AMD, glaucoma, and diabetic retinopathy. Yet also part of understanding the role of patient reported outcomes is the transition of aging-associated conditions from normal eye health to their earliest incident emergence. Thus, it is important to recognize, that even in aging, low luminance activities already play a significant role in reducing vision-targeted health related quality of life, and a health-related quality of life questionnaire reflects this trend.

## Abbreviations

AMD, age-related macular degeneration; ALSTAR, Alabama Study on Early Age-Related Macular Degeneration; AREDS, Age-Related Eye Disease Study; dB, decibels; LLQ, Low Luminance Questionnaire; logMAR, logarithm of the minimum angle of resolution; MMSE,, Mini-mental State Examination; NEI VFQ-25, National Eye Institute Visual Functioning Questionnaire (25-item version); UAB, University of Alabama at Birmingham
